# Supplementary material for: The concerted roles of FANCM and Rad52 in the protection of common fragile sites
Source: Nat Commun. 2018 Jul 18;9:2791. doi: 10.1038/s41467-018-05066-y (PMC6052092; doi:10.1038/s41467-018-05066-y)
Supplement: Supplementary file 1 — Supplementary Information [file 41467_2018_5066_MOESM1_ESM.pdf]

Supplementary Information

The concerted roles of FANCM and Rad52 in the protection of common fragile sites

Wang et al

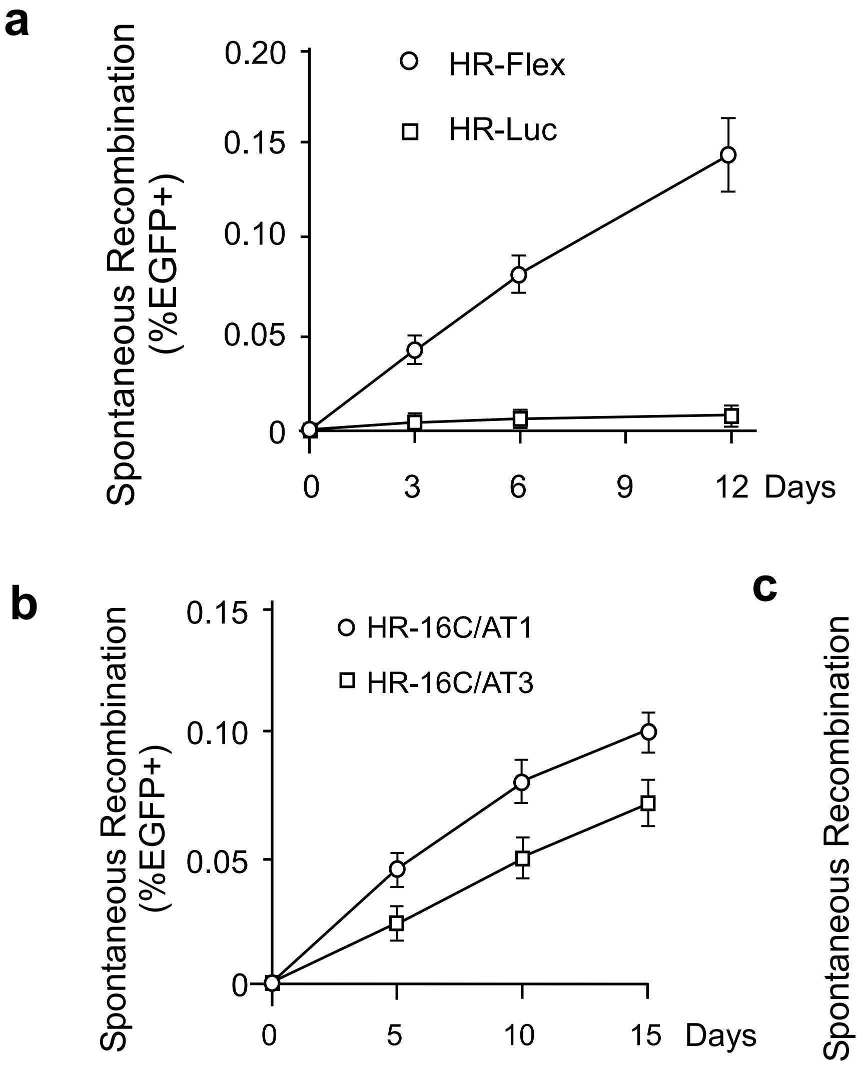

Supplementary Figure 1. AT-rich sequences derived from different CFSs induce mitotic recombination. Spontaneous recombination was examined in U2OS (HR-Flex) and U2OS (HR-Luc) cells (**a**), U2OS (HR-16C/AT1) and U2OS (HR-16C/AT3) cells (**b**), or U2OS (HR-3B/AT) cells (**c**) after culturing pre-sorted non-green cells for indicated days. Flex, 16C/AT1, 16C/AT3 and 3B/AT are AT rich sequences derived from FRA16D, FRA16C and FRA3B, respectively (see details in Methods).

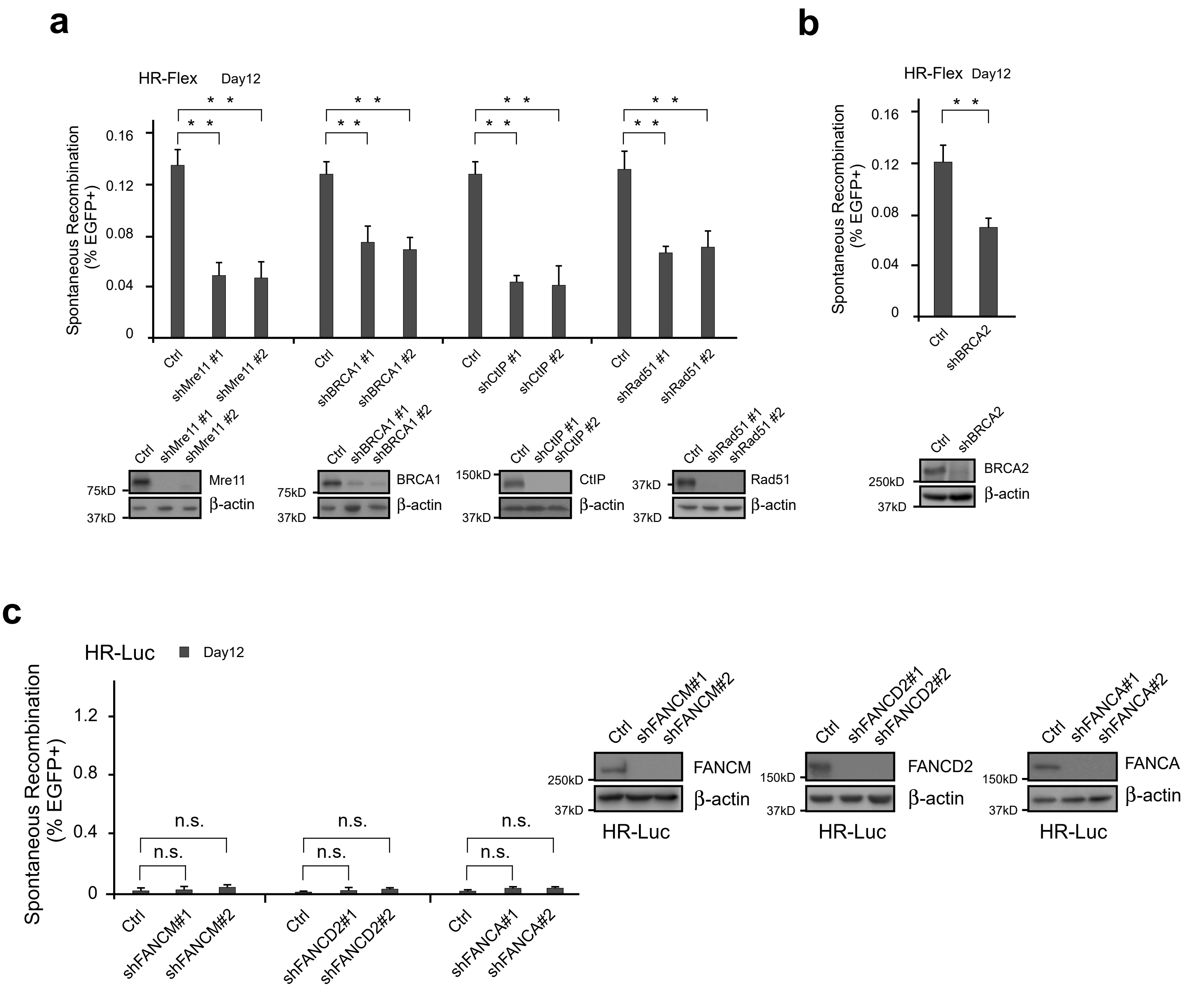

Supplementary Figure 2. Mitotic recombination is examined using HR-Flex and HR-Luc reporters when shRNAs for different repair proteins are expressed.

a and **b**, Flex1-induced mitotic recombination is suppressed by HR proteins. Spontaneous recombination was examined in U2OS (HR-Flex) 12 days after expressing two different shRNAs for indicated proteins or control vector (Ctrl). The expression of indicated proteins was examined by Western blot analysis using β-actin as a loading control. c. Mitotic recombination is examined using HR-Luc in FANCM deficient cells. Spontaneous recombination was examined in U2OS (HR-Luc) 12 days after expressing two different shRNAs for indicated proteins or control vector. The expression of indicated proteins was examined by Western blot analysis using β-actin as a loading control. Corresponding study using U2OS (HR-Flex) cells is shown in Fig.1b.

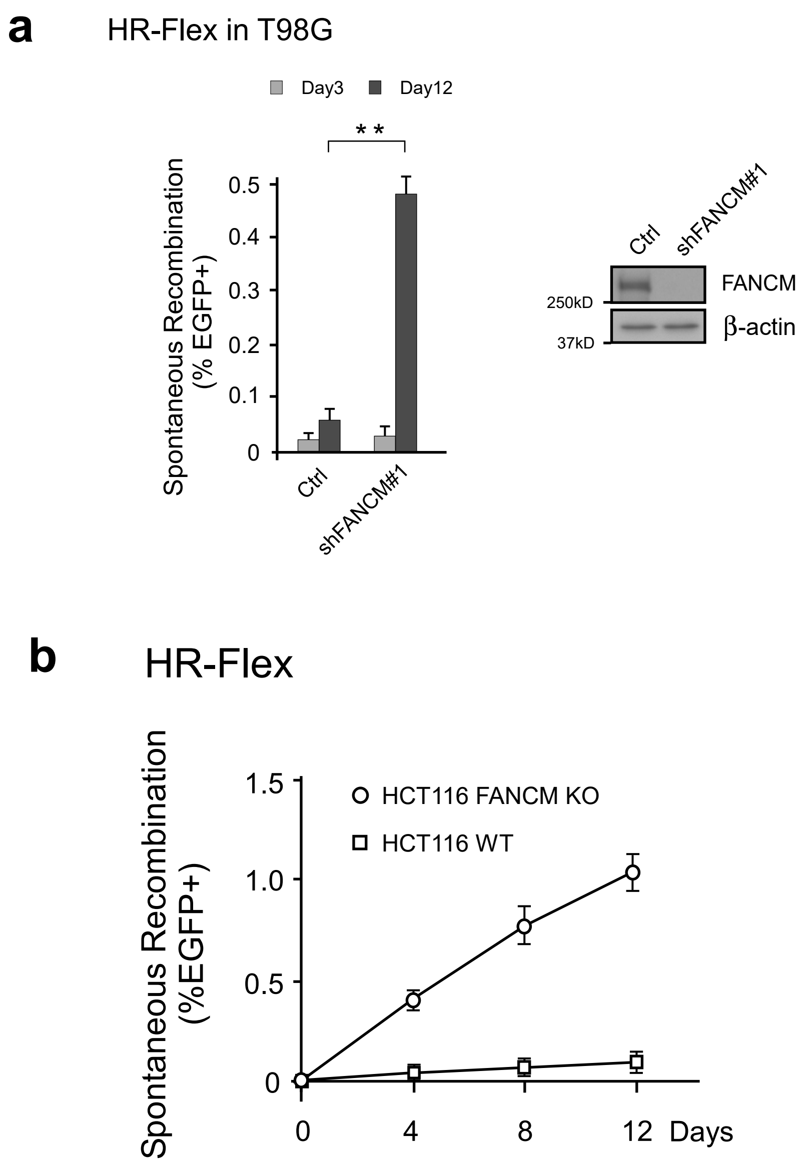

Supplementary Figure 3. Mitotic recombination at Flex1 is significantly increased when FANCM is deficient.

a, Spontaneous recombination was examined in T98G (HR-Flex) cells expressing FANCM shRNA or vector (Ctrl) after culturing pre-sorted non-green cells for indicated days.

b, Spontaneous recombination was examined in HCT116 WT or FANCM KO cells carrying HR-Flex reporter after culturing pre-sorted non-green cells for indicated days.

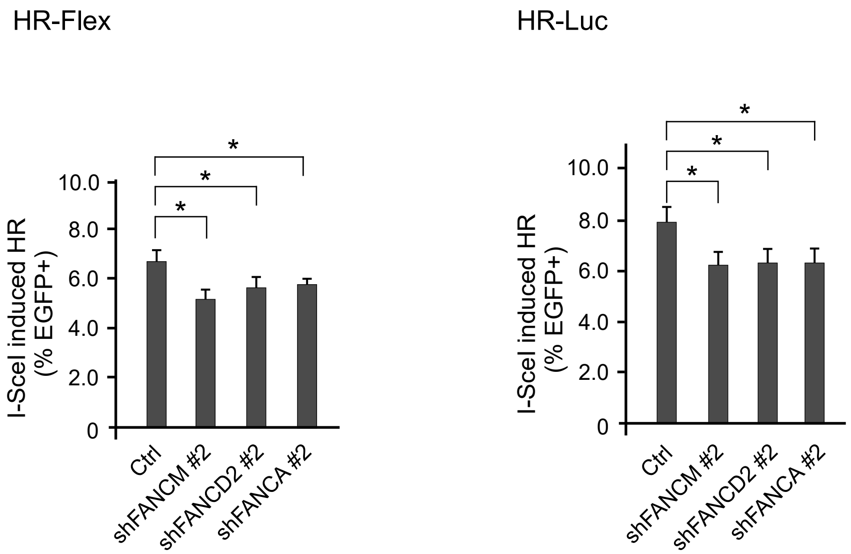

Supplementary Figure 4. Suppression of FANCM, FANCD2 and FANCA expression does not cause significant reduction of HR.
One additional shRNA (#2) was used to deplete indicated proteins in U2OS (HR-Flex) and U2OS (HR-Luc) cells. HR was assayed 5 days after viral infection of I-SceI. Related to Fig. 1f.

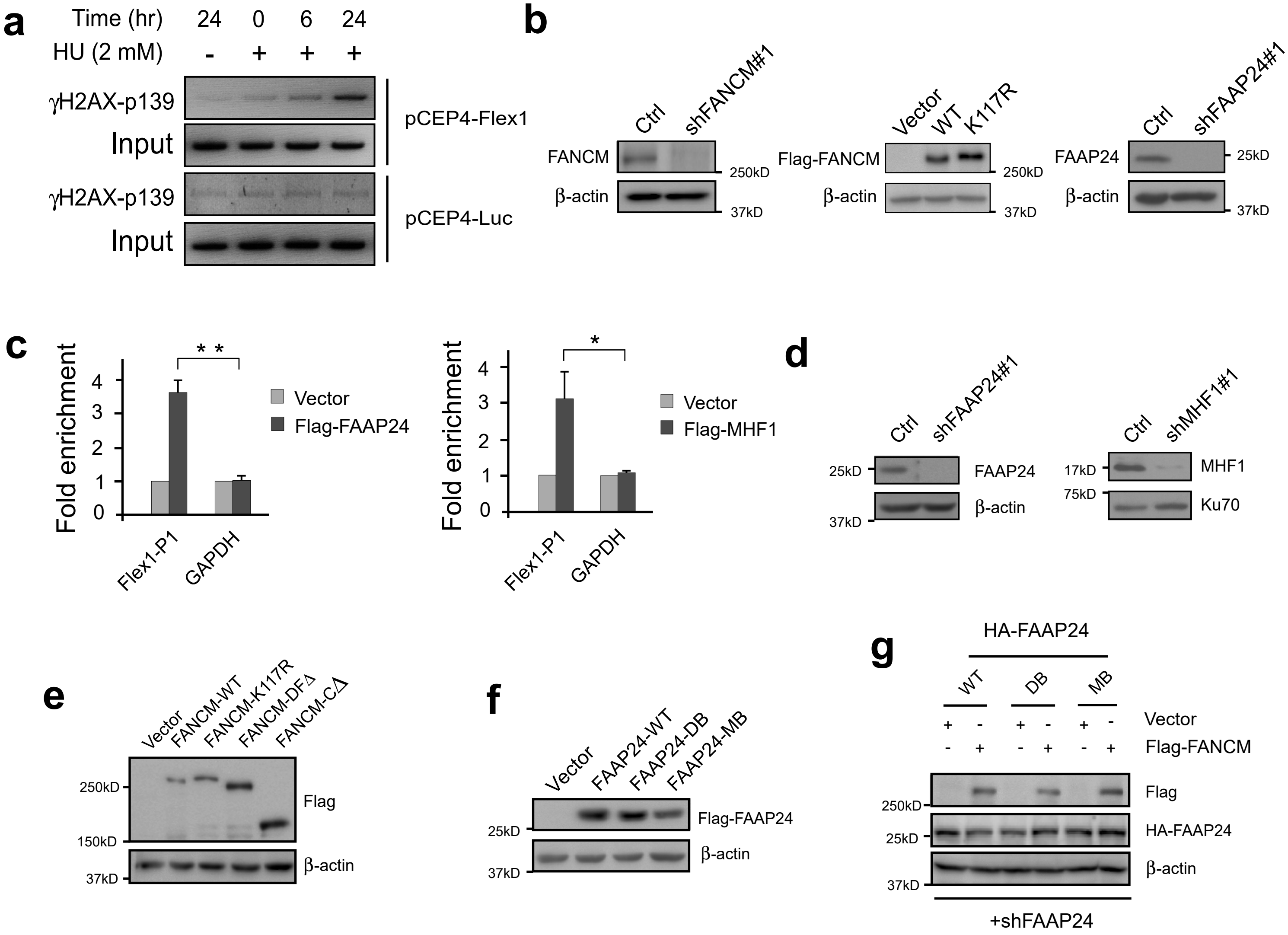

Supplementary Figure 5. FAAP24 and MHF promote FANCM binding to Flex1 to prevent DSB formation and mitotic recombination at Flex1.

a, Anti-γH2AX ChIP analysis of Flex1 or Luc was performed in U2OS cells containing pCEP4-Flex1 or pCEP4-Luc plasmids before and after HU treatment (2 mM) for 6 or 24 hr.
b, Western blots show the expression of endogenous FANCM, Flag-FANCM-WT and K117R, and endogenous FAAP24 with the cells described in Fig. 2a, using β-actin as a loading control.
c, Anti-Flag ChIP at Flex1 and GAPDH was performed in U2OS (HR-Flex) cells expressing Flag-FAAP24 (left) or Flag-MHF1 (right). RT-PCR of ChIP samples was performed using vector control, set as 1 for normalization.
d, Western blots show the expression of endogenous FAAP24 and MHF1 with the cells described in Fig. 2c and 2d. β-actin was used as a loading control.
e, Western blots show the expression of Flag-FANCM and its mutants with the cells described in Fig. 2e. β-actin was used as a loading control.
f, Western blots show the expression of Flag-FAAP24 and its mutants with the cells described in Fig. 2f (left) and 2g. β-actin was used as a loading control.
g, Western blots show the expression of Flag-FANCM and HA-FAAP24 WT or mutants with the cells described in Fig. 2f (right). β-actin was used as a loading control.

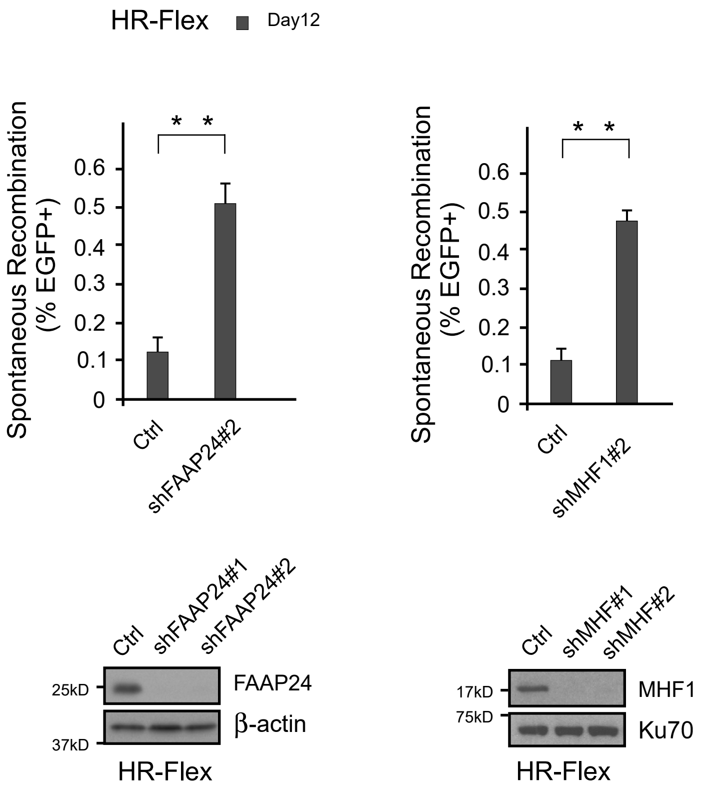

Supplementary Figure 6. FAAP24 and MHF1 are important for suppressing Flex1-induced mitotic recombination.

The expression of FAAP24 and MHF1 was suppressed by shRNA#2 in U2OS (HR-Flex) cells, and spontaneous mitotic recombination was determined after culturing pre-sorted non-green cells for 12 days. The expression of shRNA#1 and #2 for FAAP24 and MHF1 in cells used in this figure and Fig. 2d was examined by Western blot analysis.

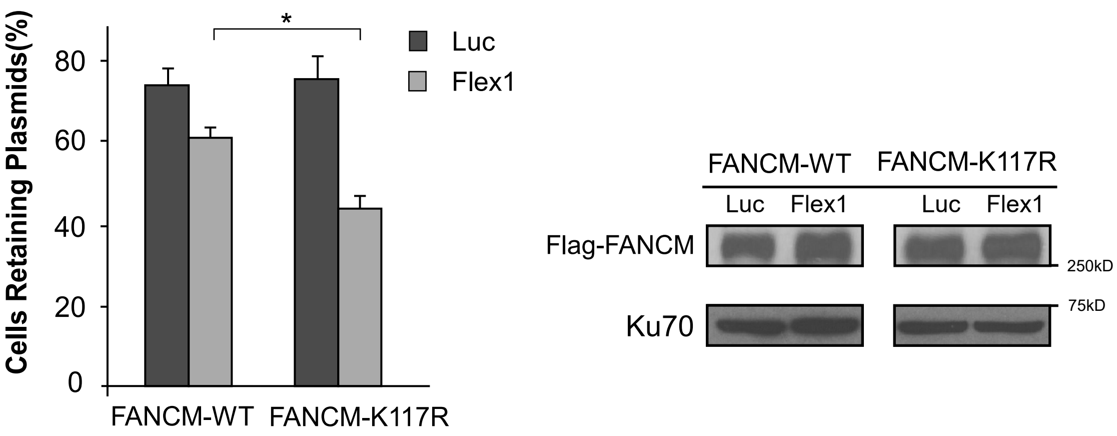

Supplementary Fiure. 7. The FANCM translocase activity is important for the stability of Flex1-containing plasmids.
Plasmid stability of pCEP4-Flex1 or pCEP4-Luc was assayed in U2OS cells expressing Flag-FANCM-WT or K117R with endogenous FANCM silenced by shRNAs (left). The expression of Flag-FANCM-WT or K117R was assayed by Western blot analysis using Ku70 as a loading control (right).

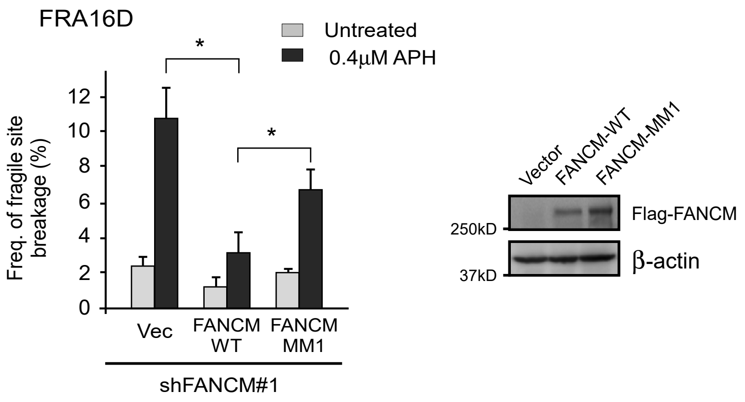

Supplementary Figure 8. CFS expression is increased in the FANCM-MM1 mutant.
Frequency of FRA16D expression in HCT116 cells was determined after expressing FANCM-WT or FANCM-MM1 alleles with endogenous FANCM silenced by shRNAs. The expression of indicated proteins was examined by Western blot analysis.

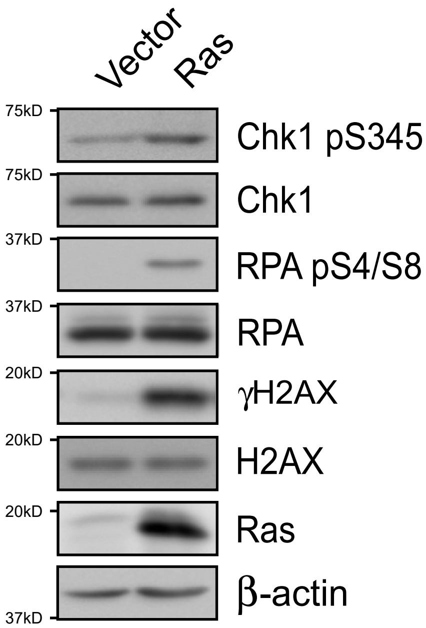

Supplementary Figure 9. Expression of H-Ras V12 (Ras) activates ATR checkpoint and induces DSB formation.
Western blot analysis of indicated proteins was performed using cell lysates purified from U2OS cells expressing vector or Ras.

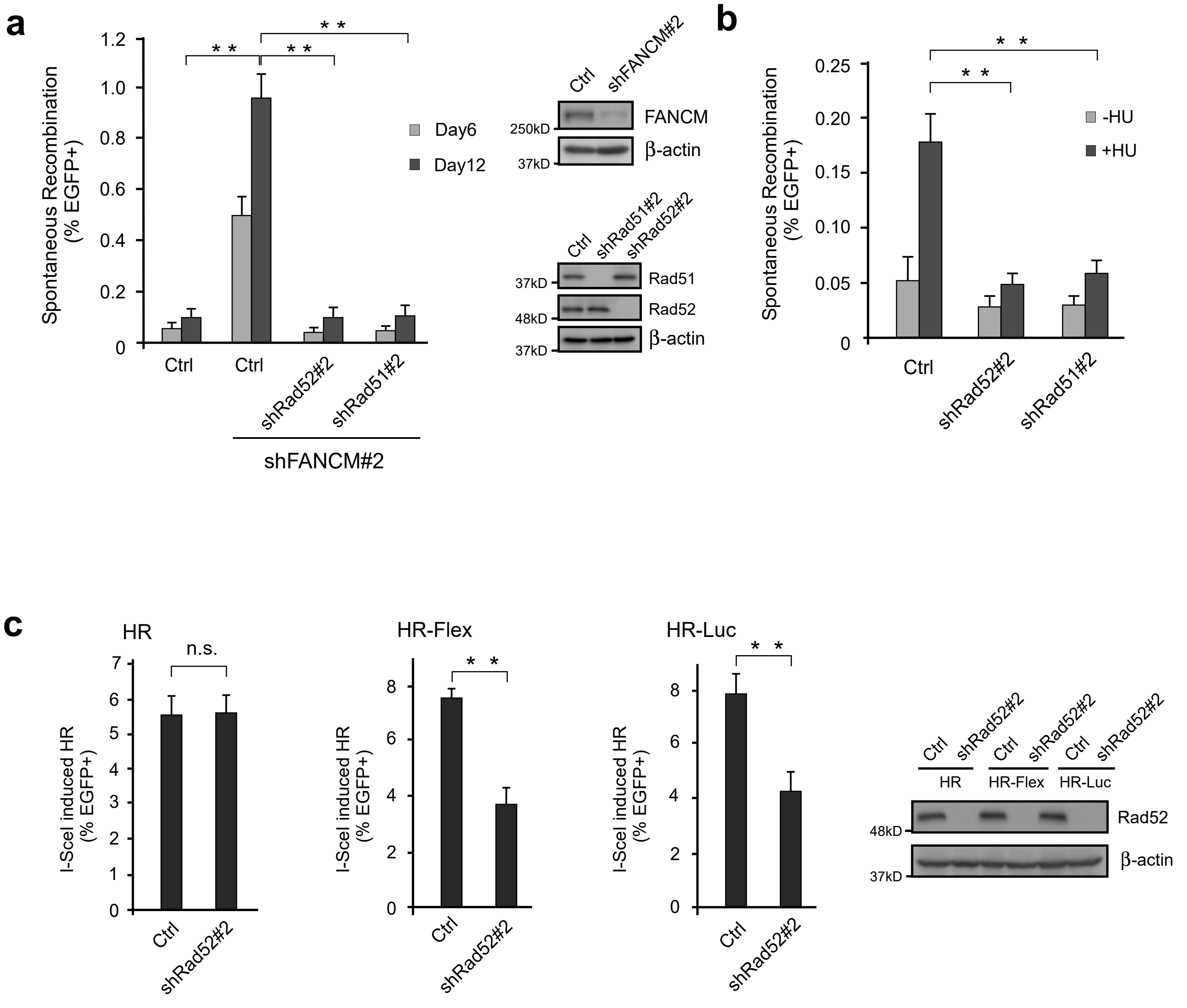

Supplementary Figure 10. Rad52 is important for repairing DSBs at Flex1.
a and **b**, Spontaneous recombination in U2OS (HR-Flex) cells expressing Rad52 shRNA#2, Rad51 shRNA#2 or control vector was examined 6 or 12 days after infection with shRNAs against FANCM or without (**a**), or 4 days after HU treatment (2 mM, 24 hr) or without (b). **c.** Rad52 shRNA#2 was expressed in U2OS (HR), U2OS (HR-Flex) and U2OS (HR-Luc) cells and I-SceI-induced HR was assayed. Western blot analysis was used to determine the expression of indicated proteins. This figure is related to Fig. 5.

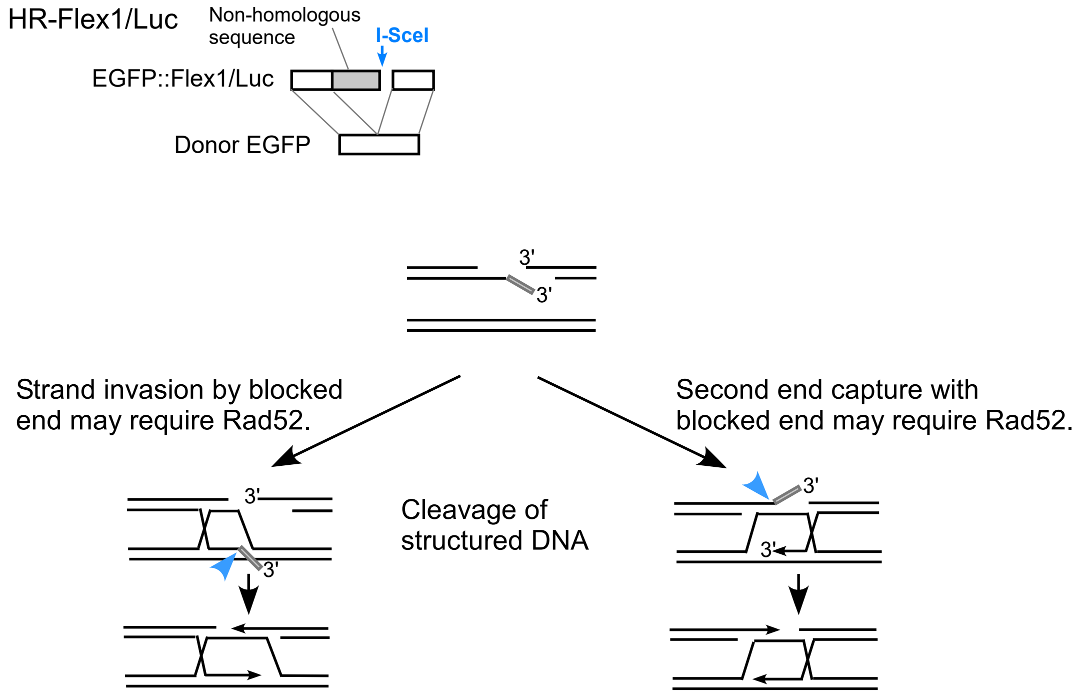

Supplementary Figure 11. Models for the role of Rad52 in repair of DSBs containing a blocked end.
A schematic drawing of HR-Flex1/Luc to show homology (white boxes) and non-homology (Flex1 or Luc, the gray box) between the EGFP::Flex1/Luc cassette and the donor EGFP cassette (top). Rad52 may be required for promoting strand invasion and/or second-end capture (bottom) when the 3' ssDNA end is blocked by a non-homologous sequence (shown as a gray box) or DNA sequences forming secondary structures (not shown, see Fig. 7). See Discussion for more details.

Figure1 b

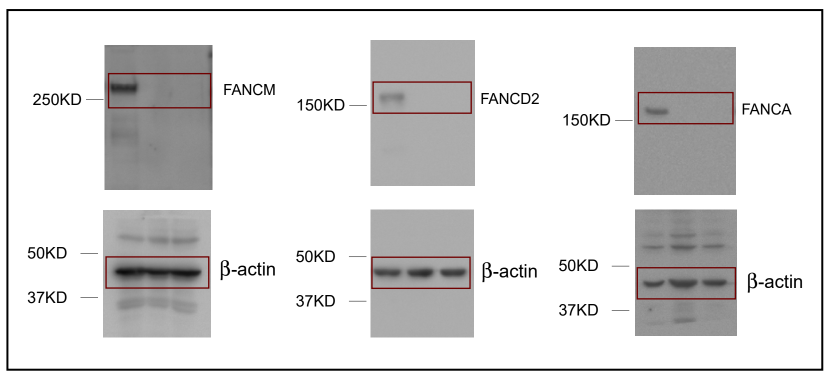

Figure1 e

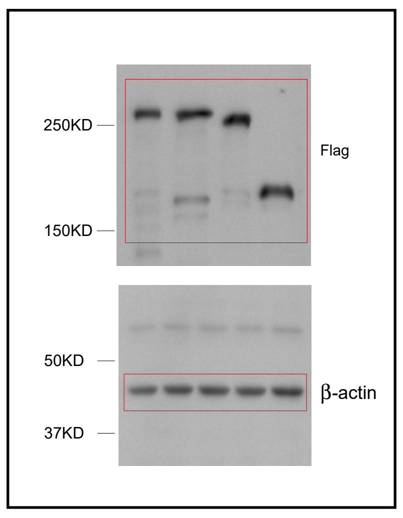

Figure2 a

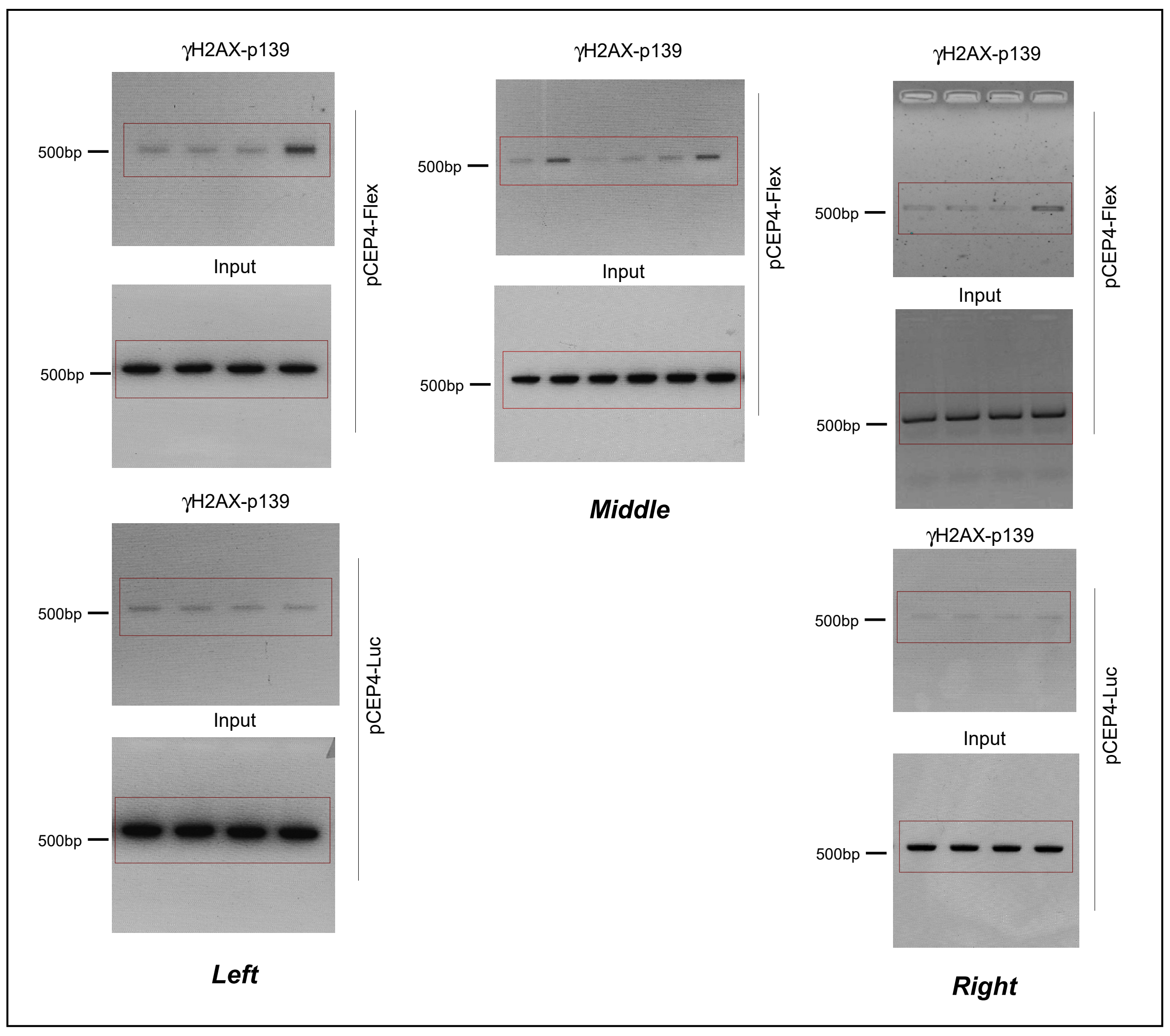

Figure1 g

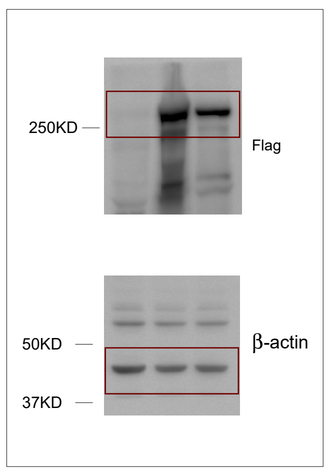

Figure1 c

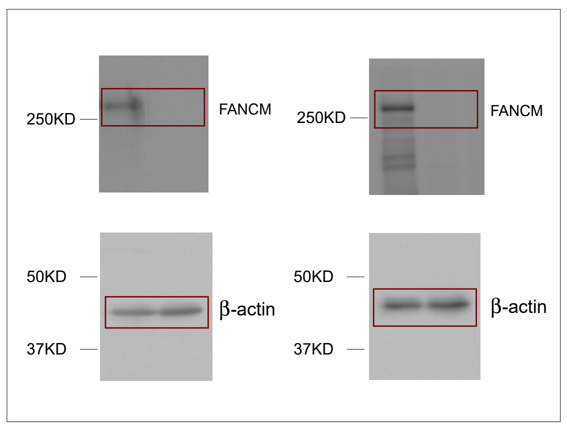

Figure 3b

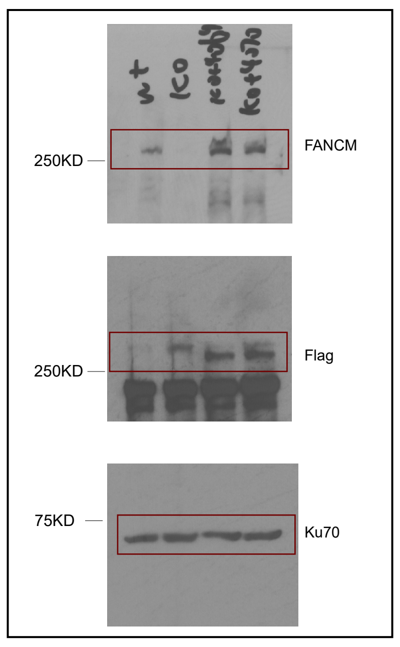

Figure 3a

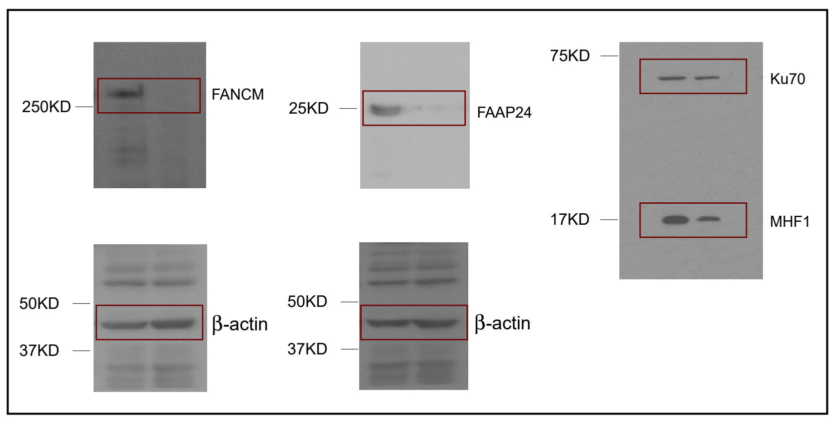

Figure 3d

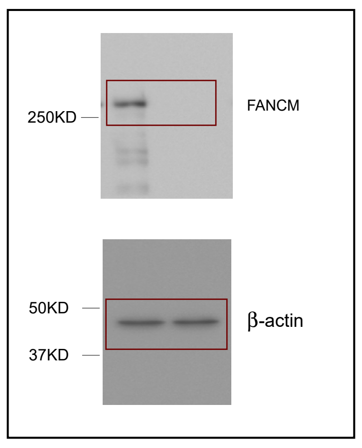

Figure 3e

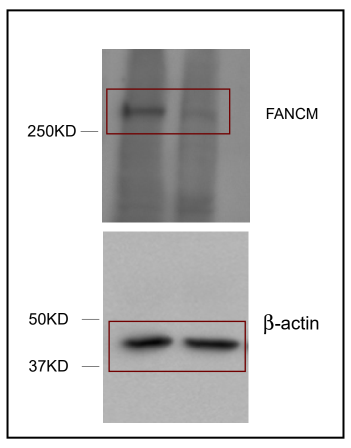

Figure 3f

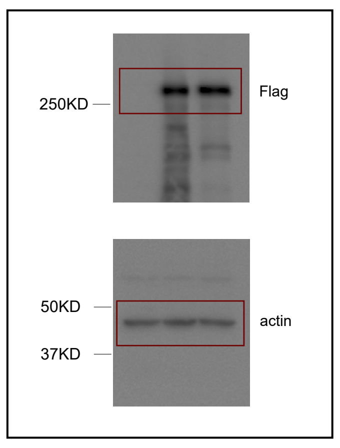

Figure 4a

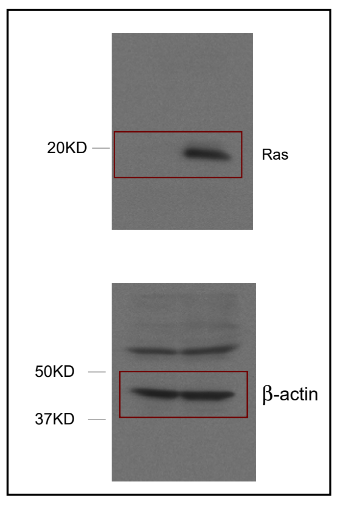

Figure 4b

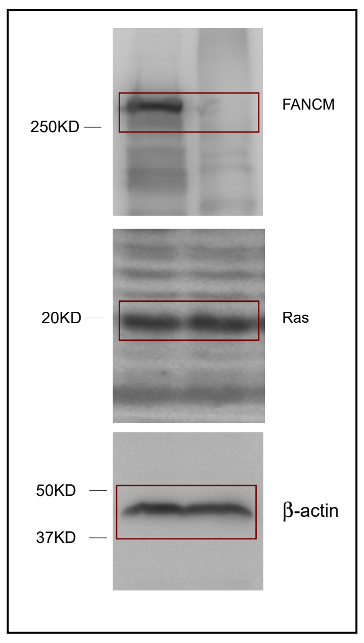

Figure 4c

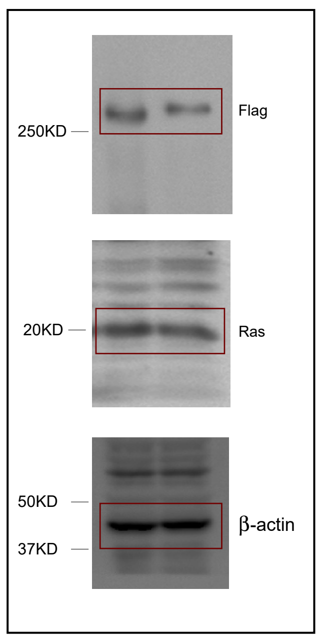

Figure 4d

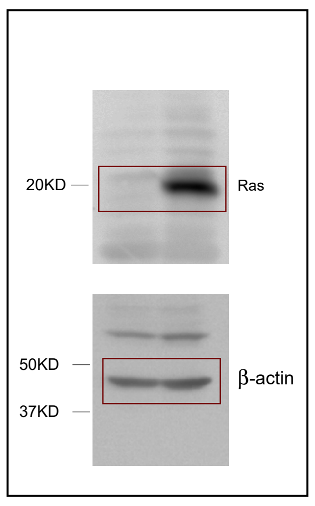

Figure 4e

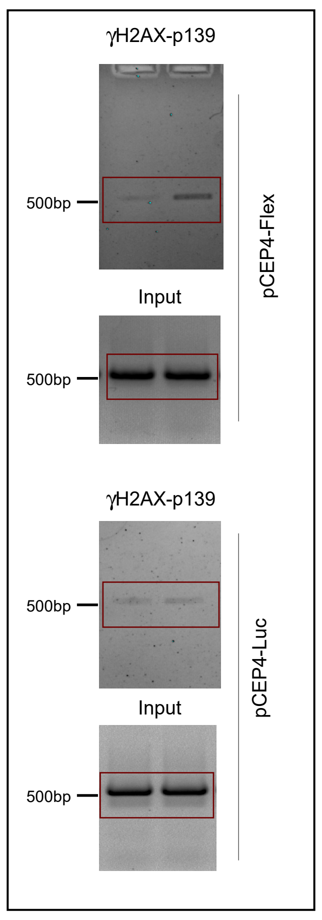

Figure 4f

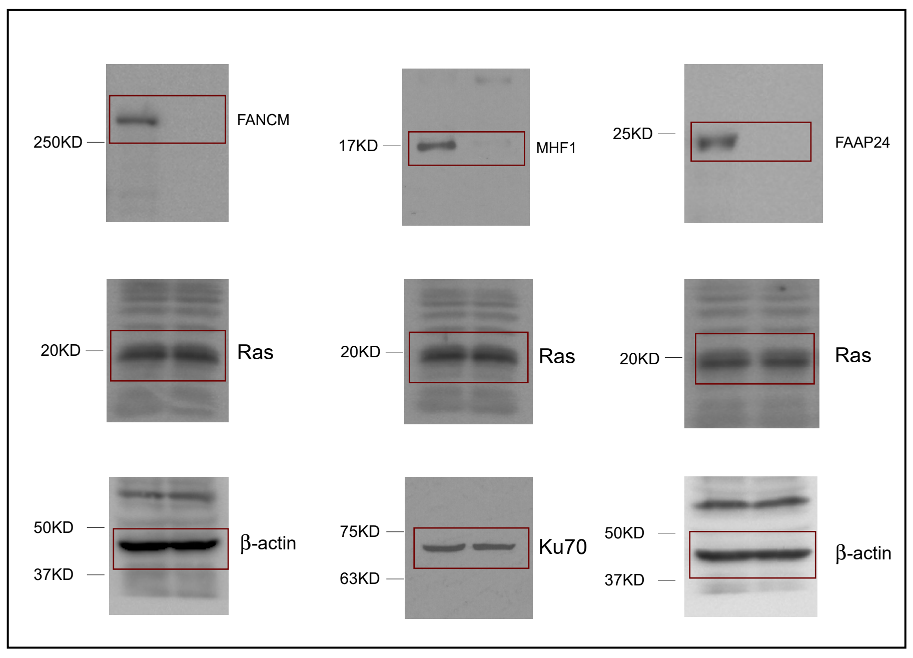

Figure 4g

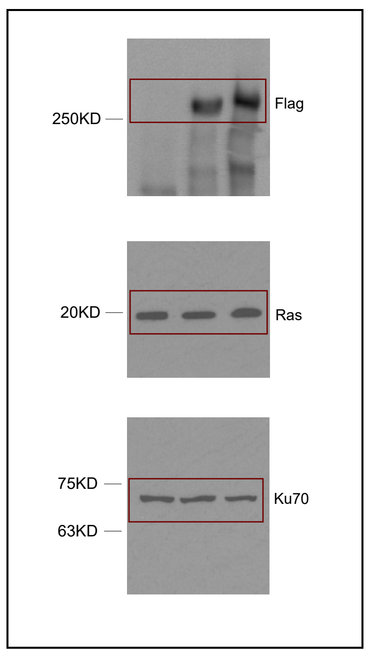

Figure 5a

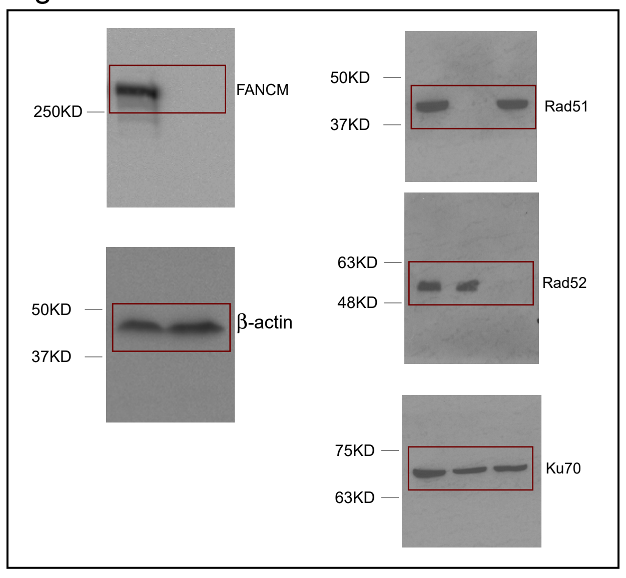

Figure 5c

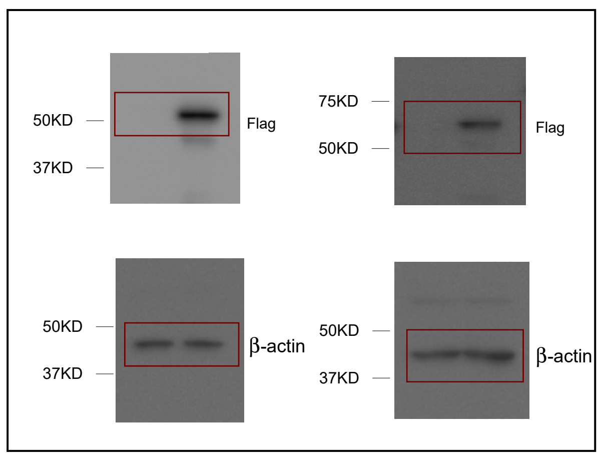

Figure 5d

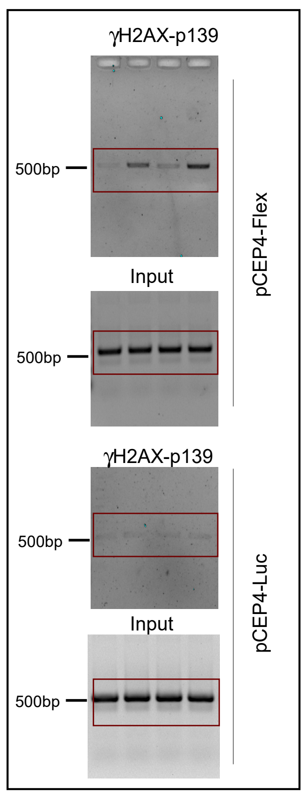

Figure 5e

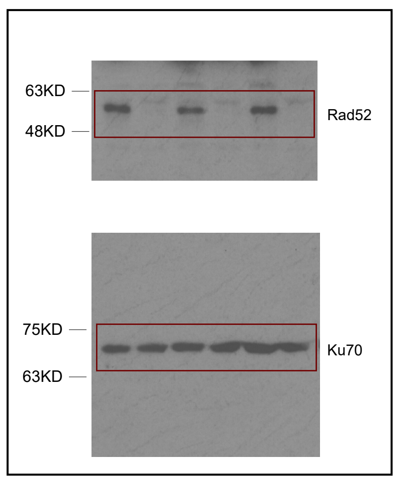

Figure 5f

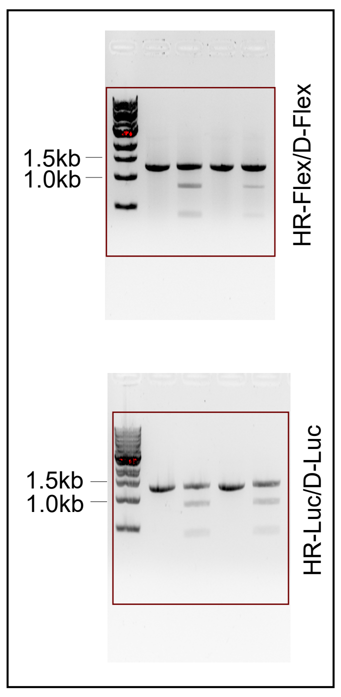

Figure 6a

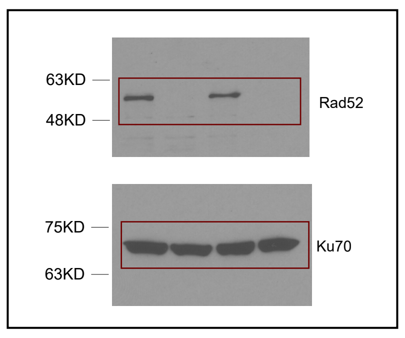

Figure 6c

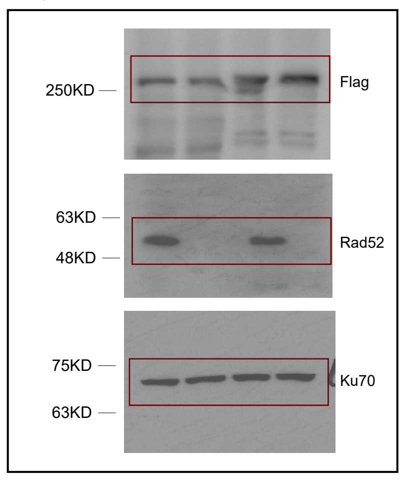

Supplementary Fig. 12. Uncropped Blots.
